# A new perspective on non-invasive diagnosis of non- alcoholic fatty liver disease: evidence integration of inflammatory and metabolic biomarkers based on a scoping review

**DOI:** 10.3389/fendo.2025.1652996

**Published:** 2025-08-28

**Authors:** Huimin Liang, Deyuan Zhang, Na Wei, Pengjiao Xi, Haize Ge, Yan Zhang

**Affiliations:** ^1^ School of Nursing, Tianjin Medical University, Tianjin, China; ^2^ Department of Nursing, Beijing Sunshine Lu-Tong Hospital of Traditional Chinese Medicine, Beijing, China; ^3^ Department of Clinical Laboratory Diagnostics, Tianjin Medical University, Tianjin, China; ^4^ Department of Clinical Laboratory, Tianjin University Central Hospital, Tianjin, China

**Keywords:** non-alchoholic fatty liver disease, inflammatory cyotokines, biomarkers, non-invasive diagnosis, scoping review

## Abstract

**Background:**

Non-alcoholic fatty liver disease (NAFLD) has become the most common chronic liver condition globally, spanning a spectrum from simple steatosis to non-alcoholic steatohepatitis (NASH) and progressive fibrosis. Inflammation and metabolic dysregulation play key roles in its pathogenesis. Accordingly, inflammatory and metabolic biomarkers have gained increasing attention as potential tools for non-invasive diagnosis and disease staging.

**Objective:**

This scoping review aimed to synthesize current evidence on the diagnostic performance of inflammatory and metabolic biomarkers for NAFLD, with a focus on their potential application in early screening and disease stratification.

**Methods:**

We systematically searched PubMed and CNKI databases for relevant peer-reviewed literature published up to August 2024. The search strategy combined MeSH terms and free-text keywords, and study selection was guided by the Population–Concept–Context (PCC) framework. Methodological quality was assessed using the Newcastle–Ottawa Scale and AHRQ criteria.

**Results:**

Fifteen eligible studies (11 case-control, 2 cohort, 1 cross-sectional, and 1 retrospective study) were included, yielding 18 candidate biomarkers. The triglyceride-glucose (TyG) index was commonly associated with early-stage NAFLD screening; cytokeratin-18 (CK18) was linked to NASH detection, while adiponectin and osteopontin (OPN) were related to liver fibrosis. Additionally, inflammatory indices such as neutrophil-to-lymphocyte ratio (NLR), monocyte-to-lymphocyte ratio (MLR), and systemic immune-inflammation index (SII) showed clinical promise due to their accessibility and low cost.

**Conclusion:**

Inflammatory and metabolic biomarkers provide valuable non-invasive insights into the diagnosis and staging of NAFLD. The integration of multiple biomarkers may enhance diagnostic accuracy and support stratified management strategies. However, further validation is needed to establish standardized thresholds and confirm clinical utility across diverse populations.

## 1 Introduction

Non-alcoholic fatty liver disease (NAFLD) is a chronic liver condition characterized by diffuse hepatic steatosis in individuals without significant alcohol consumption or other identifiable causes of liver disease. It represents a continuous spectrum ranging from simple steatosis (NAFL) to non-alcoholic steatohepatitis (NASH), liver fibrosis, cirrhosis, and even hepatocellular carcinoma (HCC). In recent years, NAFLD has become one of the most common forms of chronic liver disease globally and is closely associated with obesity, type 2 diabetes, and metabolic syndrome (1, 2).

Currently, imaging modalities such as ultrasound, CT, and MRI are commonly employed for the initial assessment of NAFLD. However, their diagnostic performance is limited by suboptimal sensitivity and specificity. Liver biopsy remains the diagnostic gold standard but is constrained in large-scale applications due to its invasiveness, sampling variability, and risk of complications (3). Consequently, there is growing interest in developing sensitive, specific, non-invasive, and reproducible biomarkers for early detection(The term “non-invasive” used in this review follows the common definition in the field of hepatology. Although venous blood sampling involves a minor invasive procedure, it does not involve tissue extraction or imaging intervention, and is typically considered non-invasive compared to liver biopsy), disease classification, and fibrosis risk assessment in NAFLD.

The pathogenesis of NAFLD involves a complex interplay of inflammatory responses, lipid metabolism disorders, oxidative stress, immune dysregulation, and cellular injury (4). Biomarkers such as inflammatory cytokines, adipokines, liver injury indicators, immune cells, and their derived inflammatory ratios have shown potential clinical value in identifying disease severity and different stages of NAFLD (5, 6).

Although previous studies have preliminarily explored these aspects, the available evidence remains fragmented, inconsistently categorized, and often lacks standardized thresholds. There is a need for systematic integration, especially to identify the most promising biomarkers and define their stage-specific clinical value in practical diagnostic pathways.

This study adopts a scoping review approach to systematically identify and summarize recent evidence on inflammatory and metabolic biomarkers related to the non-invasive diagnosis of NAFLD. The aim is to pinpoint key molecules, categorize their potential roles in early screening, disease classification, and fibrosis prediction, and provide a theoretical basis for establishing a multi-biomarker screening system to advance the precision identification and personalized management of NAFLD.

## 2 Materials and methods

### 2.1 Study design

This study was designed as a scoping review following the PRISMA-ScR (Preferred Reporting Items for Systematic Reviews and Meta-Analyses extension for Scoping Reviews) guidelines (7), The protocol of this study was registered on Open Science Framework (OSF)(https://osf.io/u4nw7). The methodological framework proposed by Daudt et al. was adopted to comprehensively examine the current status and characteristics of research on inflammatory and metabolic biomarkers in the non-invasive diagnosis of NAFLD.

### 2.2 Research questions

This review focuses on two core questions:

1. Do inflammatory or metabolic biomarkers hold diagnostic value for NAFLD?
2. Which biomarkers have shown notable performance in NAFLD screening, classification, or fibrosis risk assessment in current studies?

### 2.3 Information sources and search strategy

A comprehensive literature search was conducted across multiple databases, with tailored search terms for each platform: Chinese databases included CNKI, Wanfang, and VIP; English databases included PubMed, Web of Science, Cochrane Library, and Embase. The strategy combined subject headings and free-text terms using Boolean operators. Reference lists were also manually searched using snowballing techniques to identify additional systematic reviews. The search covered publications up to August 31, 2024. For example, the PubMed search strategy is detailed in **Table 1**. The search strategies for other databases can be found in **Supplementary Material 1**.

**Table 1:** Search strategies of Pub Med database.

Step	Search Strategy
#1	(((((((((((((((“Non-alcoholic Fatty Liver Disease”[Mesh]) OR (NAFLD [Title/Abstract])) OR (Nonalcoholic Fatty Liver Disease [Title/Abstract])) OR (Nonalcoholic Steatohepatitis [Title/Abstract])) OR (hepatic steatosis [Title/Abstract])) OR (steatohepatitis [Title/Abstract])) OR (nonalcoholic fatty liver [Title/Abstract])) OR (non-alcoholic Steatohepatitis [Title/Abstract])) OR (nonalcoholic Steatohepatitis [Title/Abstract])) OR (non-alcoholic steatosis [Title/Abstract])) OR (nonalcoholic steatosis [Title/Abstract])) OR (non-alcoholic liver steatosis [Title/Abstract])) OR (nonalcoholic liver steatosis [Title/Abstract])) OR (non-alcoholic hepatic steatosis [Title/Abstract])) OR (nonalcoholic hepatic Steatosis [Title/Abstract])) OR (nonalcoholic simple fatty liver [Title/Abstract])) OR (non-alcoholic simple fatty liver [Title/Abstract]))
#2	((((((((“Biomarkers”[Mesh]) OR Biomarker [Title/Abstract]) OR (inflammatory Marker [Title/Abstract])) OR (inflammatory markers [Title/Abstract])) OR (Serum markers [Title/Abstract])) OR (Serum marker [Title/Abstract])) OR (clinical markers [Title/Abstract])) OR (clinical marker [Title/Abstract])) OR (inflammatory factors [Title/Abstract]))
#3	#1 AND #2

### 2.4 Inclusion and exclusion criteria

Based on the PCC framework for scoping reviews (8)—Participants, Concept, and Context—the inclusion criteria were:

1. Participants: Patients with non-alcoholic fatty liver disease.
2. Concept: Studies involving inflammatory or metabolic biomarkers.
3. Context: Clinical practice or studies with original data.

Exclusion criteria were:

1. Non-original research (e.g., narrative reviews, systematic reviews, editorials, letters without original data, conference abstracts, study protocols).
2. Non-English and non-Chinese articles.
3. Duplicate publications.
4. Full text not available.

### 2.5 Study selection and data extraction

All retrieved records were imported into EndNote for deduplication. A two-stage screening process was then conducted. In the first round, two reviewers independently screened titles and abstracts. In the second round, full texts were reviewed. Disagreements were resolved through discussion or consultation with a third reviewer. Data extracted included author, publication year, country/region, study design, participant characteristics, sample size, type of biomarker, and its association with NAFLD.

### 2.6 Quality assessment

Two reviewers independently assessed the methodological quality of included studies. The Cohen’s Kappa (κ) between the reviewers was 0.80, indicating almost perfect agreement (9).Cohort and case-control studies were evaluated using the Newcastle-Ottawa Scale (NOS) (10), which includes three domains: selection, comparability, and exposure assessment, with a maximum score of 9. Scores of 0–3, 4–6, and 7–9 were interpreted as indicative of low, moderate, and high methodological quality, respectively.

Cross-sectional studies were evaluated using the quality assessment tool developed by the Agency for Healthcare Research and Quality (AHRQ) (11), comprising 11 items. Total scores of 0-3, 4-7, and 8–11 indicated low, medium, and high quality, respectively (12). Any disagreements were resolved through discussion or by a third reviewer.

## 3 Results

### 3.1 Literature search and selection

**Figure 1:**
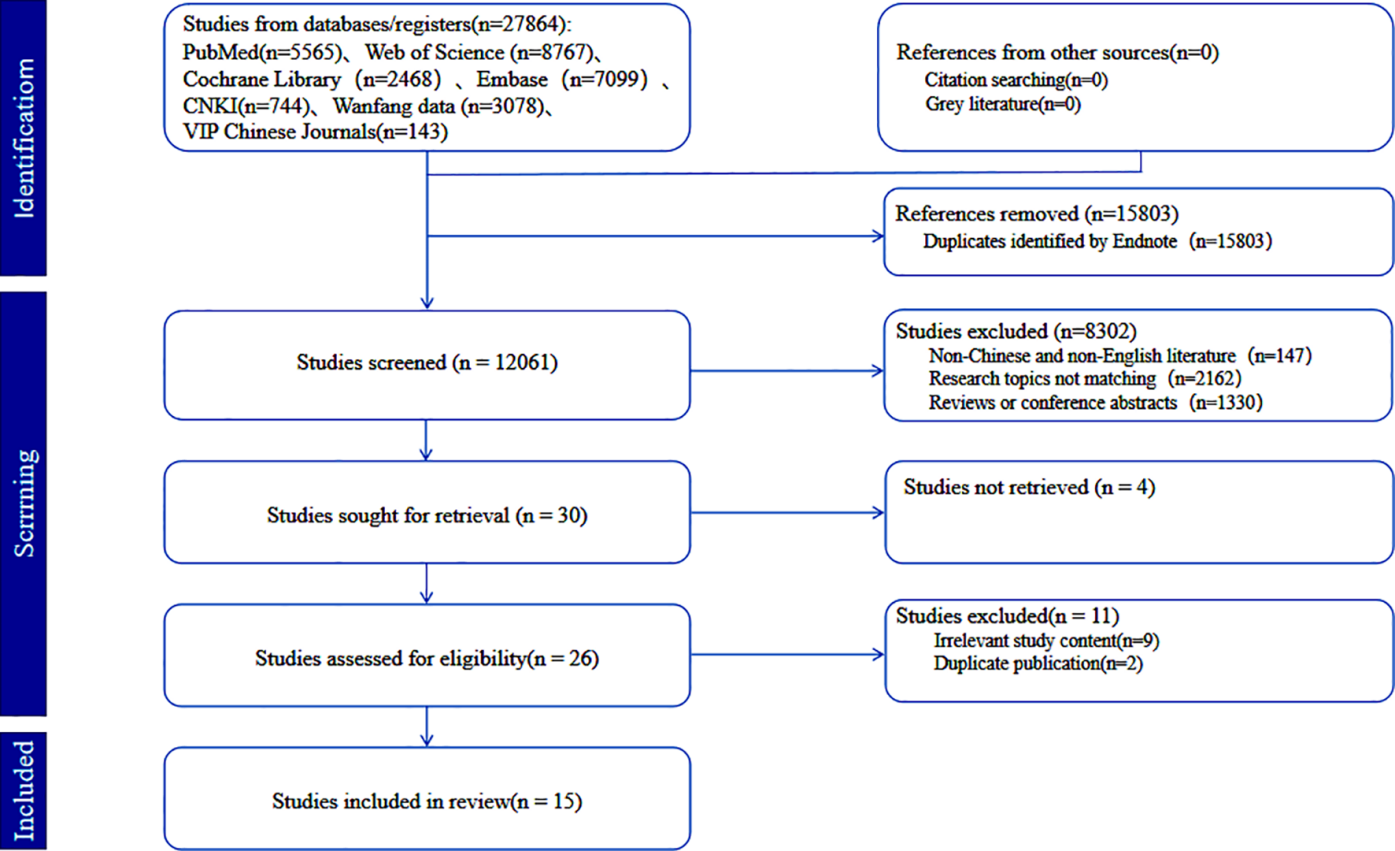
Flowchart of data selection process in accordance with PRISMA-ScR guidelines.

A total of 27,864 studies were retrieved from seven databases: CNKI (n=744), Wanfang (n=3,078), VIP (n=143), PubMed (n=5,565), Web of Science (n = 8,767), Cochrane Library (n=2,468), and Embase (n=7,099). After removing duplicates, 12,061 articles remained for screening. Based on pre-established inclusion and exclusion criteria, 15 studies were finally included (6 in Chinese and 9 in English). The study selection process is shown in the PRISMA flow diagram (13) (**Figure 1**).

### 3.2 Basic characteristics of included studies

The 15 studies were published between 2009 and 2024 and conducted in China (n =10), Turkey (n=1), Germany (n=1), Iran (n=1), Egypt (n=1), and the United States (n= 1). Most were case-control studies (n=11), with additional cohort studies (n=2), one cross-sectional study, and one retrospective study. Sample sizes ranged from 42 to 11,883 participants, including both adults and children. All included studies scored ≥7 in methodological quality assessments, indicating generally high quality. To unify the analytical logic, biomarkers were categorized into five functional groups: Inflammatory markers: CRP, IL-6, TNF-α, IL-1β, IL-18;Immune cell-derived ratios: NLR, MHR, SII, AISI, Th17/Treg;Metabolic markers: TyG index, FGF21, adiponectin, visfatin;Liver injury/inflammatory markers: CK18, OPN, PTX3, LCN2;Oxidative stress markers: Serum uric acid, hemoglobin.Detailed information is presented in **Table 2**.

**Table 2:** Basic characteristics and methodological quality assessment of included studies.

First Author (Year)	Country	Study Type	Sample Size & Population	Biomarker Category	Biomarkers	Correlation Between Biomarkers and NAFLD	Quality Score
Oruc (14) (2009)	Turkey	Case- control	100, adults	Inflammation- related biomarkers	PCT、CRP	Elevated CRP levels may assist in diagnosing NAFLD, but are not useful for distinguishing steatohepatitis from simple steatosis. PCT has no diagnostic value.	NOS 8
Li (15) (2010)	China	Case-control	348, adults	Lipid metabolism-related biomarkers	FGF21	Serum FGF21 levels are significantly elevated in NAFLD patients and may serve as a potential biomarker.	NOS 7
Jianhui Lu (16) (2013)	China	Case-control	264, adults	Inflammation- related biomarkers	IL-6	Serum IL-6 levels are elevated in NAFLD patients and may be involved in disease development.	NOS 7
Xiaoyu Ouyang (17) (2015)	China	Case-control	230, adults	Inflammation- related biomarkers	IL-6、 hs-CRP、WBC、VEGF	Serum VEGF levels are elevated in NAFLD patients and positively correlated with IL-6, hs-CRP, and WBC, suggesting inflammation is involved in disease development.	NOS8
Monika Rau (18) (2016)	Germany	Cohort study	112, adults	Inflammatory ratios of immune cells + liver injury markers	Th17/rTreg、CK18	Serum CK18 levels are significantly elevated in NAFL, NASH, and NAFLD patients, with higher levels in NASH. Th17/rTreg ratio is significantly increased in NASH and correlates positively with disease severity.	NOS 8
Jamali (19) (2016)	Iran	Case-control	54, adults	Inflammation + lipid metabolism markers	IL-6, TNF-α, APN, Visfatin	Decreased APN and increased Visfatin, IL-6, and TNF-α are positively associated with NAFLD/NASH.	NOS 8
Abdel-Razik (20)(2016)	Egypt	Case-control	1023, adults	Inflammatory ratios of immune cells	MPV, NLR	Elevated MPV and NLR ratios are valuable for predicting advanced NAFLD.	NOS 7
Glass (21)(2018)	USA	Cohort study	97, adults	Inflammation + liver injury markers	IL-8, OPN, MCP1	Levels of IL-8, OPN, and MCP1 are positively associated with fibrosis severity in NAFLD patients.	NOS 8
Shuang Tian (22)(2019)	China	Case-control	42, adolescents	Liver injury markers	LCN2, PAI1, CK18, FGF21	CK18 and FGF21 are valuable for diagnosing NAFLD; LCN2 and PAI1 may help distinguish NAFL from NASH with relatively high diagnostic value.	NOS 7
Zhao (23) (2020)	China	Case-control	8148, adults	Inflammatory ratios of immune cells	MHR, NLR, PLR, LMR	PTX-3 and TyG index are novel diagnostic biomarkers for pediatric NAFLD. Their elevation, combined with ALT, improves diagnostic efficiency.	NOS 8
Ye (24) (2021)	China	Case-control	132, children	Lipid metabolism + liver injury markers	PTX-3, TyG	Levels of IL-1β, IL-18, IL-1RA, CD4+ and CD8+ T cells are elevated in NAFLD patients. Immune factors are closely related to NAFLD/NASH classification.	NOS 8
Juan Luo (25)(2021)	China	Case-control	148, adults	Inflammation + immune cell markers	IL-1β, IL-18, IL-1R, CD4+ T, CD8+ T	Disease severity in NAFLD patients is positively correlated with IL-6, IL-8, TNF-α, and hs-CRP levels, indicating clinical monitoring value.	NOS 9
Xianhong Wang (26)(2021)	China	Case-control	180, adults	Inflammation-related biomarkers	IL-6, IL-8, TNF-α, hs-CRP	MHR is superior to NLR, PLR, and LMR as an inflammatory biomarker for NAFLD. Combining multiple indicators improves diagnostic accuracy.	NOS 8
Yajing Xian (27)(2023)	China	Cross- sectional	460, adults	Lipid metabolism + oxidative stress markers	FPG, TG, UA, HB	FPG, TG, and UA can predict NAFLD risk in middle-aged men; HB, FPG, and TG can predict risk in middle-aged women.	AHRQ 9
Bao (28) (2024)	China	Retrospective	11883, adults	Inflammatory ratios of immune cells	PLR, NLR, LMR, PNR, PMR, NAR, AGR, SII, NPAR, AISI, NLPR, SIRI	All new inflammatory biomarkers, except PLR and AGR, are significantly associated with NAFLD risk. NAR shows the highest predictive value for NAFLD and coexisting liver fibrosis.	NOS 8

NLR, Neutrophil-to-lymphocyte ratio; MHR, monocyte-to-HDL-C ratio; PLR, platelet-to-lymphocyte ratio; LMR, lymphocyte-to-monocyte ratio; IL-6, interleukins-6; interleukins-8IL-8; IL-18, interleukins-18; IL-1β, interleukins-1β; IL-1RA, interleukins-1RA; LCN2, lipocalin-2; PTX-3, pentraxin-3; PAI1, plasminogen activator inhibitor-1; CK18, cytokeratin-18; FGF21, fibroblast growth factor 21; hs-CRP, high-sensitivity C-reactive protein; WBC, white blood cell count; MPV, mean platelet volume; TNF-α, tumor necrosis factor-alpha; TyG, triglyceride-glucose index; PCT, procalcitonin; HB, hemoglobin; FPG, fasting plasma glucose; UA, uric acid; TG, triglycerides; OPN, osteopontin; MCP1, monocyte chemoattractant protein-1; APN, adiponectin; Visfatin, visfatin; Th17/rTreg, Thelper 17/regulatory T cell ratio; VEGF, vascular endothelial growth factor; PNR, platelet-to-neutrophil ratio; PMR, platelet-to-monocyte ratio; NAR, neutrophil-to-albumin ratio; NPAR, neutrophil percentage-to-albumin ratio; NLPR, neutrophil-to-lymphocyte×platelet ratio; AGR, albumin-to-globulin ratio; SII, systemic immune-inflammation index; SIRI, systemic inflammation response index; AISI, aggregate index of systemic inflammation.

### 3.3 Summary of biomarker types and diagnostic associations

Findings from the included studies suggest that inflammatory, metabolic, and liver injury biomarkers—as well as derived immune-inflammatory ratios—have potential clinical utility in identifying and stratifying different stages of NAFLD.

Inflammatory biomarkers such as IL-6, IL-1β, IL-18, TNF-α, and CRP were positively correlated with NAFLD severity, particularly IL-6 and TNF-α, which demonstrated value in disease stratification and phenotype identification.

Immune cell-derived inflammatory ratios (e.g., Th17/Treg, NLR, MHR) are accessible through routine blood tests, making them cost-effective and convenient indicators of systemic or cellular immune inflammation. Several studies found that combining these markers in predictive models enhanced diagnostic accuracy for NASH and fibrosis progression compared to traditional single biomarkers.

Metabolic biomarkers such as FGF21, TyG index, and adiponectin were closely linked to insulin resistance and hepatic steatosis, indicating their suitability for early diagnosis and high-risk population screening.

Liver injury/inflammatory biomarkers such as CK18, LCN2, PTX3, and OPN showed good performance in disease classification and fibrosis risk prediction. CK18 and LCN2 reflect hepatocellular apoptosis and tissue damage, while OPN and PTX3 are involved in inflammation-mediated fibrogenesis, aiding in the identification of NASH and advanced high-risk patients.

Oxidative stress-related biomarkers, including elevated serum uric acid and hemoglobin levels, were independently associated with NAFLD, possibly reflecting oxidative stress and metabolic burden. These may serve as useful tools for early detection in high-risk individuals.

## 4 Discussion

**Figure 2:**
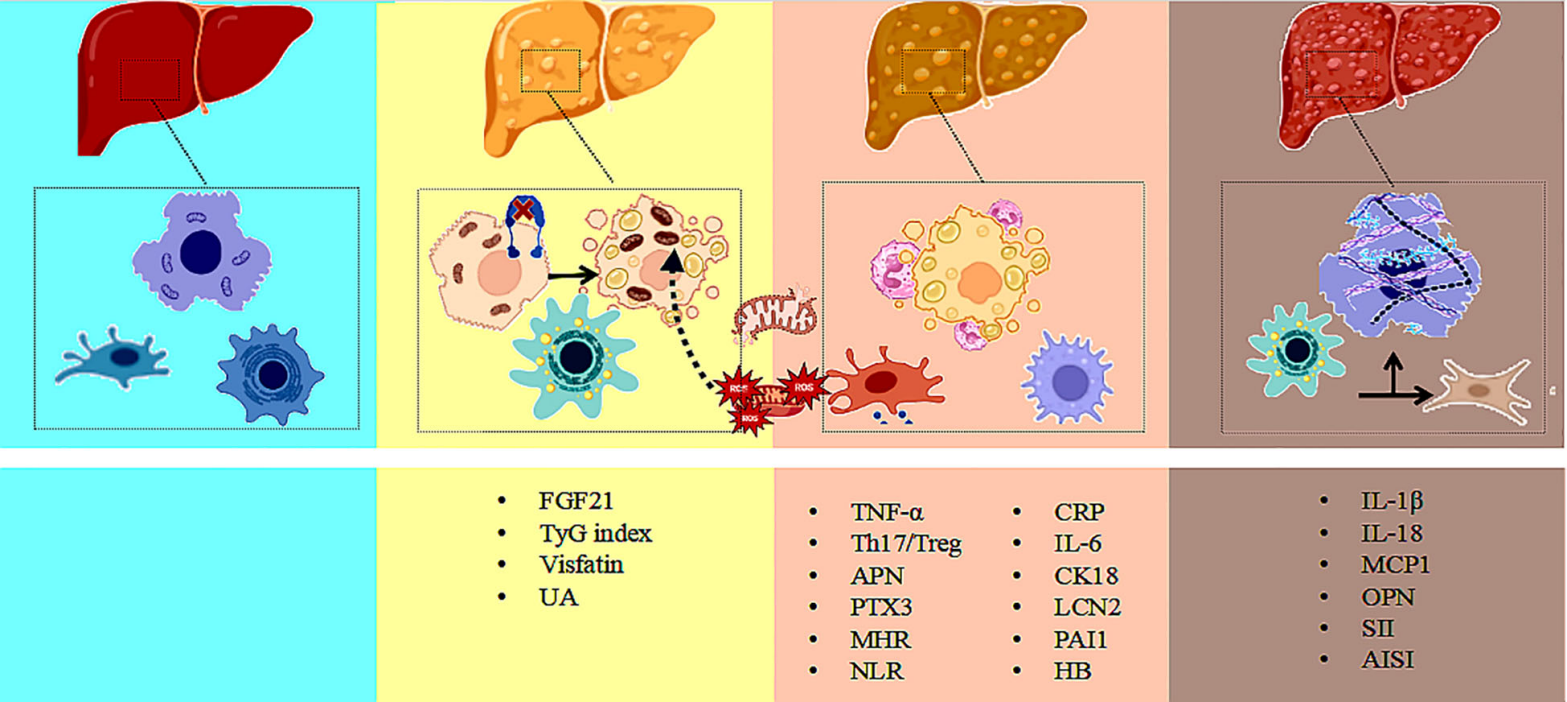
Multi-biomarker interaction network in NAFLD progression: integrating metabolic and inflammatory pathways. Illustrate the dynamic interaction network of metabolic and inflammatory biomarkers during the progression of non-alcoholic fatty liver disease, covering key biomarkers and their diagnostic applications across the NAFL, NASH, and fibrosis stages.

Nonalcoholic fatty liver disease (NAFLD) is a complex, multifactorial metabolic liver disorder. Its pathological progression typically evolves from simple steatosis (NAFL) to nonalcoholic steatohepatitis (NASH), fibrosis, cirrhosis, and eventually hepatocellular carcinoma (HCC) (29, 30). With the emergence of the “multiple-hit” hypothesis (31–33), it is now recognized that the development of NAFLD involves not only lipid accumulation (34, 35), but also a combination of mechanisms such as inflammation (36–38), insulin resistance (39, 40), genetic susceptibility (41–43) (40–42), and gut microbiota dysbiosis (44, 45). Among these, lipid metabolism disorders and subsequent cellular inflammatory responses are considered central mechanisms of NAFLD pathogenesis (46), with inflammation playing a critical role in hepatocellular injury, immune cell recruitment, and fibrosis progression (47, 48) , as illustrated in **Figure 2**.

### 4.1 Inflammation-related biomarkers

Inflammatory responses play a pivotal role in the pathogenesis of non-alcoholic fatty liver disease (NAFLD), exacerbating lipid metabolic disturbances and insulin resistance while directly promoting hepatic fat accumulation and chronic disease progression. Current evidence highlights the diagnostic significance of classical acute-phase proteins, pro-inflammatory cytokines, and immune cell-derived inflammatory ratios in disease stratification, staging assessment, and fibrosis risk prediction in NAFLD.

#### 4.1.1 Acute phase proteins

C-reactive protein (CRP) is an acute-phase protein synthesized by hepatocytes in response to inflammatory stimuli and is highly sensitive to liver inflammation in NAFLD. Studies have shown that CRP levels rise rapidly within 6–8 hours of onset and peak at 24–48 hours, correlating positively with the severity of inflammation (OR: 1.41, 95% CI: 1.31–1.51, P < 0.001) (49). High-sensitivity CRP (hs-CRP), in particular, has been significantly associated with the occurrence of non-alcoholic steatohepatitis (NASH) (OR: 1.60, 95% CI: 1.17–2.19, P = 0.003), serving as an indirect indicator of hepatic inflammatory activity (49). Although CRP lacks organ specificity, it has demonstrated strong predictive synergy in several composite diagnostic models.

#### 4.1.2 Pro-inflammatory cytokines

Tumor necrosis factor-alpha (TNF-α), primarily secreted by activated macrophages, T lymphocytes, and natural killer (NK) cells, is abundantly expressed in hepatic immune cells of patients with NAFLD (50–53). By activating the NF-κB signaling pathway, TNF-α induces hepatocyte apoptosis, promotes reactive oxygen species (ROS) production, and disrupts insulin signaling, serving as a central mediator in the inflammatory progression of NAFLD (54).Existing studies have shown that serum TNF-α levels are positively correlated with intrahepatic triglyceride levels (r = 0.28, P < 0.04) (55), suggesting potential for early screening and targeted therapy research.

Interleukin-6 (IL-6) is a pivotal cytokine regulating hepatic inflammation and metabolic dysregulation. Elevated IL-6 levels activate hepatic stellate cells via the JAK/STAT pathway, promote lipid droplet accumulation, and stimulate Kupffer cell responses, playing a key role in the transition from NAFL to NASH (56). Studies have shown that serum IL-6 levels in NAFLD patients are significantly higher than those in healthy controls(P =0.0179), and serum IL-6 levels in patients with liver fibrosis (S1, S2-3) are higher than those in non-fibrotic patients(P< 0.05) (57). Notably, IL-6 also drives Th17 cell polarization, indicating its dual role in inflammatory and immune regulation (58–60).This suggests that IL-6 levels are positively correlated with the severity of NAFLD and have the potential to serve as an early predictor of disease progression.

IL-1β and IL-18 are essential pro-inflammatory cytokines mediated by the NLRP3 inflammasome. IL-1β amplifies inflammatory cascades by inducing TNF-α and IL-6 production, while IL-18 promotes liver injury through T cell–mediated immune responses (61). Both cytokines are markedly elevated in NASH and advanced fibrosis and are closely associated with distinct NAFLD subtypes (62, 63).Furthermore, their natural antagonist, IL-1 receptor antagonist (IL-1RA), is significantly decreased in NAFLD(P< 0.05), suggesting a dysregulated inflammatory balance during disease progression (64).

#### 4.1.3 Chemokines

Monocyte chemoattractant protein-1 (MCP-1) recruits macrophages and monocytes into liver tissue during inflammation, contributing to chronic inflammation maintenance (65). MCP-1 levels positively correlate with fibrosis severity in NAFLD and may serve as a surrogate marker of hepatic fibrosis (66, 67). However, its specific role and diagnostic value in NAFLD require further evaluation.

### 4.2 Immune cells and derived inflammatory ratios

An imbalance between Th17 and Treg cells is a hallmark of immune dysregulation in NAFLD. Th17 cells secrete pro-inflammatory cytokines such as IL-17, contributing to hepatic inflammation and lipid accumulation, whereas Treg cells release IL-10 and exert immunosuppressive and anti-inflammatory effects (68, 69). Studies have shown that the Th17/Treg ratio is significantly elevated in patients with NAFLD and is closely associated with insulin resistance and liver injury, indicating its potential as an early warning signal for NASH risk (18).

Peripheral counts of neutrophils, monocytes, and lymphocytes, as well as their derived ratios, serve as accessible indicators of systemic inflammation and have demonstrated potential in early NAFLD screening (68, 70). Neutrophil infiltration is a characteristic feature of NASH, and elevated neutrophil counts have been identified as an independent risk factor for NAFLD (71). As key components of innate immunity, intermediate monocyte subsets (CD14++CD16+) are significantly increased in NAFLD and may serve as potential predictive markers (72).

CD4+ T lymphocytes play a central role in adaptive immune regulation and can differentiate into Th1, Th2, Th17, and Treg subsets, coordinating both pro- and anti-inflammatory responses in liver tissue (73). Impaired proliferation and activation of CD4+ and CD8+ T cells may compromise immune surveillance, thereby facilitating NAFLD progression (74). Lymphocyte aggregation correlates positively with lobular inflammation and fibrosis staging, suggesting a potential bridging role in the transition from NAFL to NASH (75).

Composite inflammatory indices derived from routine hematological and biochemical parameters have gained attention for their simplicity, low cost, and applicability in large-scale non-invasive screening of NAFLD. These indices reflect subclinical systemic inflammation and include the neutrophil-to-lymphocyte ratio (NLR), monocyte-to-HDL ratio (MHR), systemic immune-inflammation index (SII), and aggregate index of systemic inflammation (AISI). NLR is markedly elevated in patients with moderate-to-severe NAFLD and NASH (76); MHR reflects the balance between immune activation and anti-inflammatory capacity (77); and SII and AISI, as integrative markers, have demonstrated strong discriminatory power in disease stratification and fibrosis prediction (78). These scoring systems help reduce the bias of single biomarkers and support early risk stratification and individualized therapeutic decision-making.

### 4.3 Lipid metabolism-related biomarkers

The liver plays a central role in lipid metabolism, encompassing fatty acid synthesis, transport, and oxidation. Disruptions in lipid metabolic homeostasis constitute a foundational mechanism in the pathogenesis of NAFLD and interact bidirectionally with insulin resistance and oxidative stress, leading to lipotoxicity and chronic inflammation (79). Accordingly, lipid metabolism–related biomarkers hold potential clinical value in early screening and identification of high-risk individuals.

Fibroblast growth factor 21 (FGF21), primarily secreted by hepatocytes, regulates lipogenesis, energy metabolism, and insulin sensitivity (80). In patients with NAFLD, elevated FGF21 levels are thought to represent a compensatory response to metabolic stress, particularly endoplasmic reticulum stress (81). A recent study demonstrated promising diagnostic performance of FGF21 for NAFLD, with an AUC of 0.832 (95% CI: 0.77–0.886, P < 0.001), suggesting its potential utility as an adjunctive biomarker (82).

Adiponectin, an adipokine with anti-inflammatory, antioxidant, and anti-fibrotic properties, is markedly reduced during the progression from NAFLD to NASH (83). Hypoadiponectinemia is closely linked to insulin resistance and increased cardiovascular risk (84). In a comparative analysis, adiponectin outperformed several metabolic markers in differentiating NAFLD subtypes, with an AUC of 0.643–0.644 (95% CI: 0.089–0.345, P < 0.001) (85).

Visfatin, secreted primarily by visceral adipose tissue, exhibits insulin-mimetic properties by modulating glucose metabolism and insulin signaling pathways (86). Reduced visfatin levels may exacerbate insulin resistance and contribute to NAFLD progression, underscoring its potential as an indicator of metabolic dysregulation (87).

The triglyceride-glucose (TyG) index, derived from fasting plasma glucose and triglyceride levels, was initially developed to evaluate insulin resistance (88, 89). Multiple studies have demonstrated its superior diagnostic accuracy for NAFLD compared with traditional predictors (90, 91). The TyG index reflects underlying mechanisms of glucotoxicity and lipotoxicity that drive insulin resistance and hepatic fat accumulation (92). It has shown robust predictive capability for NAFLD, with an AUC of 0.782 (95% CI: 0.773–0.790), sensitivity of 72.2%, and specificity of 70.5% (93).

### 4.4 Combined markers of liver injury and inflammation

The progression of NAFLD involves hepatocellular apoptosis, tissue remodeling, and persistent inflammation. Certain serum biomarkers reflect both hepatic injury and systemic inflammation, thereby offering dual diagnostic value for disease classification and fibrosis risk prediction.

Cytokeratin-18 (CK-18) is a specific marker of hepatocyte apoptosis, released into circulation upon membrane disruption (94). Elevated serum CK-18 levels help distinguish NAFL from NASH, with the M30 fragment showing good diagnostic performance (AUC = 0.750, 95% CI: 0.714-0.787). When the CK-18 concentration exceeds 375 U/L, the diagnostic performance improves (AUC = 0.79), with a sensitivity of 81.5%, specificity of 65.0%, positive predictive value (PPV) of 80.8%, and negative predictive value (NPV) of 43.1% (P < 0.0001) (95).

Lipocalin-2 (LCN2), a glycoprotein secreted primarily by neutrophils, is upregulated in hepatic injury and inflammatory states. Induced by endotoxins, IL-6, and IL-1β, LCN2 contributes to neutrophil infiltration and upregulation of chemokine receptor CXCR2 (96–98). Its serum levels are significantly elevated in NASH and exhibit high diagnostic accuracy: for NAFLD, a cutoff of >57.57 ng/mL yields 76.47% sensitivity and 100% specificity; for NASH, a cutoff of >84.485 ng/mL yields 84.62% sensitivity and 80.95% specificity. The corresponding AUC values are 0.882 (95% CI: 0.745–0.961) and 0.868 (95% CI: 0.708–0.959), respectively (22).

Pentraxin 3 (PTX3), an acute-phase protein with immunomodulatory properties, is structurally similar to C-reactive protein (CRP) but exhibits higher specificity. Elevated PTX3 levels in NAFLD are associated with steatosis severity and hepatic enzyme abnormalities (99) (100). In pediatric NAFLD, PTX3 has a diagnostic cutoff of 3.03 U/L for NASH (sensitivity: 89%, specificity: 86%) (100). In adults, plasma PTX3 levels correlate significantly with NAFLD activity score, fibrosis stage, and steatosis grade (r = 0.659, P < 0.001; r = 0.354, P < 0.01; r = 0.455, P < 0.001). A cutoff of 2.45 ng/mL yields 91.1% sensitivity and 71.4% specificity for diagnosing NASH in adults (101).

Osteopontin (OPN), a glycoprotein secreted by adipose tissue macrophages, is closely associated with advanced hepatic fibrosis. OPN expression increases with fibrosis severity and is significantly elevated in patients with stage ≥F3 NAFLD (P < 0.001), supporting its potential use as a biomarker for progressive disease (102, 103).

Plasminogen activator inhibitor-1 (PAI-1), synthesized by hepatocytes and visceral adipose tissue, contributes to extracellular matrix accumulation and hepatic stellate cell activation. Elevated PAI-1 levels are associated with obesity, dyslipidemia, and inflammation, and promote fibrogenic progression in NAFLD (104, 105). Notably, PAI-1 demonstrates high diagnostic accuracy for NASH (106); a serum level >11.60 ng/mL yields an AUC of 0.954 (95% CI: 0.884–0.988), with 100% sensitivity and 90% specificity (107).

### 4.5 Oxidative stress-related biomarkers

Oxidative stress is one of the key mechanisms driving the progression of non-alcoholic fatty liver disease (NAFLD) from metabolic dysfunction to tissue damage and liver fibrosis. The excessive production of reactive oxygen species (ROS), induced by metabolic toxicity, fatty acid peroxidation, and mitochondrial dysfunction, activates Kupffer cells, disrupts hepatocyte membrane integrity, and triggers chronic inflammation (108). Therefore, oxidative stress-related biomarkers serve as intermediate indicators of the pathogenesis of NAFLD, with potential diagnostic and prognostic value.

Uric acid has a dual biological role: at low concentrations, it acts as an antioxidant, while at higher concentrations, it becomes a pro-oxidative and pro-inflammatory mediator (109). Hyperuricemia is closely associated with the onset and progression of NAFLD, contributing to liver damage by activating immune responses, promoting insulin resistance, and exacerbating oxidative stress (110). Studies have shown that even within the normal range, uric acid levels are an independent risk factor for NAFLD (OR (95% CI): 1.46 (1.17-1.82) to 2.13 (1.42-3.18)) (111). Uric acid is not only related to the severity of liver damage in NAFLD but also holds potential as a predictive biomarker for disease progression. A study in Korea found a positive correlation between serum uric acid levels and the five-year incidence of NAFLD, suggesting its potential as an early diagnostic marker (112). Furthermore, a meta-analysis showed that the SUA threshold for NAFLD was 308 μmol/L, with a sensitivity of 94.12% (71.3-99.9) and a specificity of 70.6% (44.0-89.7) (113). However, the diagnostic performance of uric acid may vary among different populations, and further analysis of its cut-off value variability is needed to ensure its accuracy and reliability in broad clinical applications.

The role of iron metabolism biomarkers in NAFLD is also significant, particularly with hemoglobin (Hb) and ferritin. Ferritin catalyzes the production of free radicals, which may exacerbate oxidative stress (114). Animal studies have demonstrated that iron overload in NASH models significantly affects the progression of NAFLD, and iron-reducing treatment has shown protective effects against NASH (115). In NAFLD patients, Hb is considered an important predictor of liver fibrosis, especially in lean NAFLD (BMI < 25 kg/m²), where Hb is the only independent predictor (116). Thus, Hb can serve as a serum biomarker for NAFLD patients with normal weight, assisting in diagnosis and disease progression assessment.

## 5 Conclusions and limitations

With the rising global prevalence of nonalcoholic fatty liver disease (NAFLD), there is an increasing clinical demand for noninvasive diagnostic tools capable of early detection and progression stratification. Inflammation and metabolic dysregulation, as core mechanisms underlying NAFLD, have prompted extensive research into related biomarkers for noninvasive assessment. Based on a systematic scoping review approach, this study summarizes the diagnostic potential and strength of evidence for various inflammatory and metabolic biomarkers across different clinical stages of NAFLD—including early screening, NASH differentiation, fibrosis evaluation, and complication prediction.

From the integrated findings, multiple biomarkers exhibit distinct advantages at specific diagnostic stages. For example, the TyG index shows superior performance in early screening; CK18 is highly specific for NASH diagnosis; and OPN demonstrates significant value in fibrosis assessment. Composite inflammatory ratios (e.g., NLR, MLR, AISI, SII) have shown strong translational potential due to their simplicity and accessibility. Meanwhile, metabolic markers such as adiponectin and FGF21 exhibit dynamic correlations with disease severity, supporting their potential role in disease monitoring and intervention evaluation.

Overall, the clinical value of inflammation- and metabolism-related biomarkers in the noninvasive diagnosis of NAFLD is becoming increasingly evident, particularly in the development of predictive models and risk scoring systems. However, limitations persist in current studies, including sample heterogeneity, inconsistency in diagnostic criteria, and a lack of prospective validation. Future research should focus on large-scale, multicenter studies and mechanistic investigations to facilitate the transition of high-value biomarkers from “research indicators” to “clinical tools,” ultimately advancing precision diagnosis and treatment of NAFLD.

Among the 15 studies included in this scoping review, 10 were conducted in China. This geographical concentration may introduce regional bias and limit the generalizability of the findings to Western or multi-ethnic populations. It reflects the active research landscape in East Asia, particularly in China, where hospital-based studies on non-invasive biomarkers for NAFLD are prominent. Although several key biomarkers showed consistent diagnostic trends across studies, supporting their potential clinical value, the evidence drawn mainly from a single region should be interpreted with caution. Further validation in multi-center cohorts with geographic and ethnic diversity is needed to assess the global applicability of these biomarkers. In addition, this review did not systematically search grey literature databases or clinical trial registries, which may lead to a risk of publication bias. Future reviews should broaden information sources and incorporate grey literature and registered trial data to improve the comprehensiveness and representativeness of the findings.

## Data Availability

Publicly available datasets were analyzed in this study. This data can be found here: https://osf.io/u4nw7.
