# Supplementary material for: A new perspective on non-invasive diagnosis of non- alcoholic fatty liver disease: evidence integration of inflammatory and metabolic biomarkers based on a scoping review
Source: Front Endocrinol (Lausanne). 2025 Aug 28;16:1652996. doi: 10.3389/fendo.2025.1652996 (PMC12422897; doi:10.3389/fendo.2025.1652996)
Supplement: Supplementary file 1 [file Table1.docx]

# Supplementary Material 1. Search Strategies for All Databases (as of August 31, 2024)

## PubMed

| Step | Search Strategy |
| --- | --- |
| # 1 | ((((((((((((((((("Non-alcoholic Fatty Liver Disease"[Mesh]) OR(NAFLD [Title/Abstract])) OR (Nonalcoholic Fatty Liver Disease [Title/Abstract])) OR (Nonalcoholic Steatohepatitis [Title/Abstract])) OR (hepatic steatosis [Title/Abstract])) OR (steatohepatitis [Title/Abstract])) OR (nonalcoholic fatty liver [Title/Abstract])) OR(non-alcoholic Steatohepatitis [Title/Abstract])) OR(nonalcoholic Steatohepatitis [Title/Abstract]))OR (non-alcoholic steatosis [Title/Abstract)) OR(nonalcoholic steatosis [Title/Abstract])) OR (non-alcoholic liver steatosis [Title/Abstract])) OR(nonalcoholic liver steatosis [Title/Abstract])) OR (non-alcoholic hepatic steatosis [Title/Abstract])) OR (nonalcoholic hepatic Steatosis [Title/Abstract])) OR (nonalcoholic simple fatty liver [Title/Abstract)) OR non-alcoholic simple fatty liver [Title/Abstract]) |
| # 2 | (((((((("Biomarkers"[Mesh]) OR Biomarker [Title/Abstract]) OR (inflammatory Marker [Title/Abstract])) OR (inflammatory markers [Title/Abstract])) OR (Serum markers [Title/Abstract])) OR (Serum marker [Title/Abstract])) OR (clinical markers [Title/Abstract])) OR (clinical marker [Title/Abstract]) OR (inflammatory factors [Title/Abstract])) |
| # 3 | #1 AND #2 |

## Web of Science

| Step | Search Strategy |
| --- | --- |
| # 1 | TS=(Non-alcoholic Fatty Liver Disease OR NAFLD OR Nonalcoholic Fatty Liver Disease OR Nonalcoholic Steatohepatitis OR hepatic steatosis OR steatohepatitis OR nonalcoholic fatty liver OR non-alcoholic Steatohepatitis OR nonalcoholic Steatohepatitis OR non-alcoholic steatosis OR nonalcoholic steatosis OR non-alcoholic liver steatosis OR nonalcoholic liver steatosis OR non-alcoholic hepatic steatosis OR nonalcoholic hepatic Steatosis OR nonalcoholic simple fatty liver OR non-alcoholic simple fatty liver) |
| # 2 | TS=(Biomarkers OR Biomarker OR inflammatory Marker OR inflammatory markers OR Serum markers OR Serum marker OR clinical markers OR clinical marker OR inflammatory factors) |
| # 3 | #1 AND #2 |

## Cochrane Library

| Step | Search Strategy |
| --- | --- |
| # 1 | [mh "Non-alcoholic Fatty Liver Disease"] OR ("NAFLD":ti,ab,kw) OR ("Nonalcoholic Fatty Liver Disease":ti,ab,kw) OR ("Nonalcoholic Steatohepatitis":ti,ab,kw) OR ("hepatic steatosis":ti,ab,kw) OR ("steatohepatitis":ti,ab,kw) OR ("nonalcoholic fatty liver":ti,ab,kw) OR ("non-alcoholic Steatohepatitis":ti,ab,kw) OR ("nonalcoholic Steatohepatitis":ti,ab,kw) OR ("non-alcoholic steatosis":ti,ab,kw) OR ("nonalcoholic steatosis":ti,ab,kw) OR ("non-alcoholic liver steatosis":ti,ab,kw) OR ("nonalcoholic liver steatosis":ti,ab,kw) OR ("non-alcoholic hepatic steatosis":ti,ab,kw) OR ("nonalcoholic hepatic Steatosis":ti,ab,kw) OR ("nonalcoholic simple fatty liver":ti,ab,kw) OR ("non-alcoholic simple fatty liver":ti,ab,kw) |
| # 2 | [mh "Biological Markers"] OR ("Biomarker*":ti,ab,kw) OR ("inflammatory Marker*":ti,ab,kw) OR ("Serum marker*":ti,ab,kw) OR ("clinical marker*":ti,ab,kw) OR ("inflammatory factor*":ti,ab,kw) |
| # 3 | #1 AND #2 |

## Embase

| Step | Search Strategy |
| --- | --- |
| # 1 | 'non alcoholic fatty liver'/exp OR 'nafld':ti,ab,kw OR 'nonalcoholic fatty liver disease':ti,ab,kw OR 'nonalcoholic steatohepatitis':ti,ab,kw OR 'hepatic steatosis':ti,ab,kw OR 'steatohepatitis':ti,ab,kw OR 'nonalcoholic fatty liver':ti,ab,kw OR 'non alcoholic steatohepatitis':ti,ab,kw OR 'nonalcoholic steatohepatitis':ti,ab,kw OR 'non alcoholic steatosis':ti,ab,kw OR 'nonalcoholic steatosis':ti,ab,kw OR 'non alcoholic liver steatosis':ti,ab,kw OR 'nonalcoholic liver steatosis':ti,ab,kw OR 'non alcoholic hepatic steatosis':ti,ab,kw OR 'nonalcoholic hepatic steatosis':ti,ab,kw OR 'nonalcoholic simple fatty liver':ti,ab,kw OR 'non alcoholic simple fatty liver':ti,ab,kw |
| # 2 | 'biological marker'/exp OR 'biomarker*':ti,ab,kw OR 'inflammatory marker*':ti,ab,kw OR 'serum marker*':ti,ab,kw OR 'clinical marker*':ti,ab,kw OR 'inflammatory factor*':ti,ab,kw |
| # 3 | #1 AND #2 |

## CNKI（中国知网）

| Step | Search Strategy |
| --- | --- |
| # 1 | SU = ('Non-alcoholic fatty liver' + 'Non-alcoholic fatty liver disease' + 'Nonalcoholic fatty liver disease' + 'Non-alcoholic steatohepatitis' + 'Fatty liver' + 'Steatohepatitis' + 'Hepatic steatosis' + 'Liver steatosis') |
| # 2 | SU = ('Biomarkers' + 'Biological markers' + 'Serum biomarkers' + 'Serum markers' + 'Inflammatory biomarkers' + 'Inflammatory markers' + 'Inflammatory cytokines' + 'Clinical biomarkers' + 'Diagnostic biomarkers') |
| # 3 | #1 AND #2 |

## Wanfang（万方）

| Step | Search Strategy |
| --- | --- |
| # 1 | Subject:(Non-alcoholic fatty liver OR Non-alcoholic fatty liver disease OR Nonalcoholic fatty liver disease OR Non-alcoholic steatohepatitis OR Fatty liver OR Steatohepatitis OR Hepatic steatosis OR Liver steatosis) |
| # 2 | Subject:(Biomarkers OR Biological markers OR Serum biomarkers OR Serum markers OR Inflammatory biomarkers OR Inflammatory markers OR Inflammatory cytokines OR Clinical biomarkers OR Diagnostic biomarkers) |
| # 3 | #1 AND #2 |

## VIP（维普）

| Step | Search Strategy |
| --- | --- |
| # 1 | M=(Non-alcoholic fatty liver + Non-alcoholic fatty liver disease + Nonalcoholic fatty liver disease + Non-alcoholic steatohepatitis + Fatty liver + Steatohepatitis + Hepatic steatosis + Liver steatosis) |
| # 2 | M=(Biomarkers + Biological markers + Serum biomarkers + Serum markers + Inflammatory biomarkers + Inflammatory markers + Inflammatory cytokines + Clinical biomarkers + Diagnostic biomarkers) |
| # 3 | #1 AND #2 |
